# Testosterone as Potential Effective Therapy in Treatment of Obesity in Men with Testosterone Deficiency: A Review

**DOI:** 10.2174/157339912799424573

**Published:** 2012-03

**Authors:** Farid Saad, Antonio Aversa, Andrea M Isidori, Louis J Gooren

**Affiliations:** 1 Bayer Pharma, Scientific Affairs Men’s Healthcare, Berlin, Germany and Gulf Medical University School of Medicine, Ajman, UAE; 2 Medical Pathophysiology and Endocrinology Section, Department of Experimental Medicine, Sapienza Università di Roma, Rome, Italy; 3 Department of Endocrinology, VUmc, Amsterdam, the Netherlands

**Keywords:** Obesity, metabolic syndrome, testosterone, diabetes mellitus, weight reduction, drug safety.

## Abstract

**Objective:**

Obesity negatively affects human health. Limiting food intake, while producing some weight loss, results in reduction of lean body mass. Combined with moderate exercise it produces significant weight loss, maintains lean body mass and improves insulin sensitivity, but appears difficult to adhere to. Bariatric surgery is clinically effective for severely obese individuals compared with non-surgical interventions, but has limitations. Clinical and pre-clinical studies have implicated a role for testosterone (T) in the patho-physiology of obesity.

**Methods:**

Evidence Acquisition and Synthesis: A literature search in PubMed on the role of T in counteracting obesity and its complications.

**Results:**

Obesity per se impairs testicular T biosynthesis. Furthermore, lower-than-normal T levels increase accumulation of fat depots, particularly abdominal (visceral) fat. This fat distribution is associated with development of metabolic syndrome (MetS) and its sequels, namely type 2 diabetes mellitus (T2DM) and cardiovascular disease (CVD). T treatment reverses fat accumulation with significant improvement in lean body mass, insulin sensitivity and biochemical profiles of cardiovascular risk. The contribution of T to combating obesity in hypogonadal men remains largely unknown to medical professionals managing patients with obesity and metabolic syndrome. Many physicians associate T treatment in men with risks for prostate malignancy and CVD. These beliefs are not supported by recent insights.

**Conclusion:**

While overall treatment of obesity is unsuccessful, T treatment of hypogonadal men may be effective, also because it improves mood, energy, reduces fatigue and may motivate men to adhere to diet and exercise regimens designed to combat obesity.

## INTRODUCTION

Obesity is reaching epidemic proportions worldwide with profound impact on health resulting in reduced quality of life, early death. If obesity will continue to rise, it will undoubtedly increase health care costs and impede economic progress and national well being of many countries [1, 2]. Deposition of excess fatty acids (FAs) into fat cells in the form of triglycerides (TGs) is the biochemical basis of obesity, thus any imbalance in food intake and energy utilization may result in obesity. This homeostasis is complex and is regulated by a host of metabolic and endocrine factors which are poorly understood. Obesity contributes to pathologies, such as the metabolic syndrome (MetS), cardiovascular disease (CVD), type 2 diabetes mellitus (T2DM), hypertension, endothelial dysfunction [ED] and testosterone deficiency (hypogonadism) [TD]. Obese individuals often exhibit characteristics of MetS [3] with increased body mass index (BMI), waist circumference (WC) and waist-to-hip ratio (WHR). 

Differences in gender distribution of body fat are well recognized [4] and in men, fat accumulates in the abdominal regions (both subcutaneous and intra-abdominal or visceral fat depots) and generally exhibits larger visceral fat depot relative to (premenopausal) women [5, 6]. In premenopausal women, a larger proportion of fat is stored in peripheral fat depots such as breasts, hips and thighs. Not surprisingly, sex steroids have been shown to contribute to this sex difference. Fat distribution (a large visceral fat depot) is a strong predictor of CVD and of the development of T2DM, and is also linked to a negative biochemical cardiovascular risk profile [7]. 

## ROLE OF TESTOSTERONE IN ETIOLOGY, PATHOPHYSIOLOGY AND TREATMENT OF MALE OBESITY 

In addition to its role as a critical steroid hormone in reproductive and sexual functioning, T is an important signaling molecule in regulating energy utilization and multiple cellular metabolic pathways, including nitrogen retention and regulation of adipogenesis [3, 8, 9]. Considerable evidence exists with regard to the role of T in increasing and maintaining muscle mass and reducing fat mass and therefore regulation of body composition [10]. This suggests that TD may contribute to the etiology of obesity and, in case of hypogonadism, T treatment may turn out to be beneficial in managing obesity, also in combination with exercise and diet [11, 12]. As shown in Fig. (**1**), testosterone treatment improves body composition. The potential of testosterone to improve the metabolic situation in hypogonadal has met with reservation. For many decades, T was perceived by the medical community to play a role in the development of prostate cancer (PCa) [13], and was thought of as a risk factor for CVD [14]. The higher prevalence of coronary heart disease in men, compared with women, was attributed to T, being the most obvious gender difference. Recent clinical studies have proven both assumptions inaccurate. There is no compelling evidence that T is a significant factor in the etiology of CVD [14] and of PCa [13], but once these unwarranted perceptions had become common they are difficult to discredit. In this review, we summarize the role of T in the etiology and treatment of obesity in men with TD from three perspectives: 1) evidence from epidemiological and observational studies, 2) evidence from androgen deprivation therapy (ADT), mainly in men undergoing treatment for PCa, and 3) evidence from T treatment of men with TD. Rather than to advocate testosterone in the large field of anti-obesity treatment, the aim is to define more precisely a role for testosterone in the treatment of obesity and its associated conditions in men with TD which has so far not received serious consideration. Naturally, the potential benefits must be balanced against its (long-term) risks.

## RELATIONSHIP BETWEEN TESTOSTERONE AND OBESITY

TD (hypogonadism) in men is defined as a serum T less than 12 nmol/L [15] combined with a set of clinical signs and symptoms, as outlined by several professional societies [16]. Several studies have demonstrated an inverse relationship between indicators of obesity (body mass index, waist circumference, a reliable indicator of visceral obesity), and T levels over all age groups [3, 17, 18]. Obesity contributes to onset of T2DM, dyslipidemia, hypertension and, therefore, MetS. An inverse association between the severity of features of MetS, T2DM and plasma T has been previously reported [19]. In one study, this association was independent of age and body mass index [20], underlining the complexity of the relationship between testosterone and obesity [21]. This became also apparent from another study that verified the prevalence of low testosterone levels in male T2DM patients, related to variations in BMI, waist circumference, neuropathy, triglycerides, CRP, glucose, insulin and HOMA-IR, but no increase of silent myocardial ischemia or peripheral arterial disease was established [22]. This is supported by other studies linking low testosterone, cardiovascular risk and insulin resistance (for review [23]). 

Although age is associated with the prevalence of MetS, young men with features of the MetS exhibit reduced T levels [24, 25] and T treatment in these individuals positively affects weight reduction, with concomitant reduction in insulin resistance (IR). The exact pathophysiological mechanisms responsible for reduced T levels in obesity remain under investigation [3], however, hyperinsulinemia is shown to suppress serum T levels [26]. T levels are reduced in men with T2DM [17], with an inverse association between T levels and glycosylated hemoglobin (HbA_1c_) [27] and this occurs independently of medications, such as statins [28]. In men with low plasma T, the likelihood of T2DM is increased and several large prospective studies have shown that low T levels predict development of T2DM in men. Low levels of T are associated with a decreased lean body mass, and relative muscle mass is inversely associated with insulin resistance and prediabetes [29]. Epidemiological evidence from several longitudinal population studies shows that low T is an independent risk factor for the development of both MetS and T2DM [17, 28, 30], stroke or transient ischemic attacks [31]. A systematic review and meta-analysis of cross-sectional studies indicated that T level was significantly lower in men with T2DM (mean difference, -76.6 ng/dL, 95% confidence interval [CI], -99.4 to -53.6). Prospective studies have shown that men with higher T levels (range, 449.6-605.2 ng/dL) had a 42% lower risk of T2DM (RR, 0.58, 95% CI, 0.39 to 0.87). Reduced levels of total T and sex hormone binding globulin (SHBG) (associated with IR) were both independent risk factors in middle-aged men who later developed T2DM, according to The Massachusetts Male Aging Study (MMAS) and the Multiple Risk Factor Intervention Trial (MRFIT) [32, 33].

In The Rancho-Bernardo Study, a significant inverse correlation was demonstrated between baseline total T, with long-term (8-year follow-up) fasting glucose and insulin levels as well as glucose intolerance [34]. In men, endogenous testosterone concentrations were inversely related to mortality due to cardiovascular disease and all causes [35]. The authors concluded that low testosterone may be a predictive marker for those at high risk of cardiovascular disease. The Tromso study nuanced these observations [36]. In this study, there was a significant increase in all-cause mortality risk for men with free testosterone in the lowest quartile (<158 pmol/L) compared with the higher quartiles after age adjustment hazard ratios (HR 1.24, 95% confidence interval, CI 1.01-1.53) and after multivariate adjustments (HR 1.24, 95% CI 1.01-1.54). Total testosterone was not associated with mortality risk. Likewise, there were no significant changes in risk for first-ever MI across different total or free testosterone levels. Men with free testosterone levels in the lowest quartile had a 24% increased risk of all-cause mortality.

Recently, the Third National Health and Nutrition survey (NHANES III) in a population of 1,413 men after adjustment for age, race/ethnicity and adiposity, showed that those men initially in the lowest tertile of either free or bioavailable (but not total) T were approximately four times more likely to have T2DM compared to those in the third tertile [37]. Collectively, these findings support those reported by the MMAS in that the risk is independent of adiposity [17, 30, 31]. In a recent review and meta-analysis of endogenous testosterone and mortality in men, Araujo *et al.* concluded that low endogenous testosterone levels are associated with increased risk of all-cause and cardiovascular death in community-based studies of men, but with considerable between-study heterogeneity, which was related to study and subject characteristics, which suggests that effects are driven by differences between cohorts (e.g. in underlying health status) [38].

## THE VICIOUS CIRCLE OF LOW TESTOSTERONE AND OBESITY

Synthesis of SHBG and T is a highly correlative biochemical process. Hyperinsulinism, associated with adiposity, suppresses synthesis of SHBG and thus, levels of circulating T [39, 40]. In addition, insulin [41] and leptin [42] exert suppressive effects on testicular steroidogenesis and may contribute to further disruption of pulse amplitude of luteinizing hormone (LH) diminishing stimulation of the testicular steroidogenesis. Further, conversion of T to estradiol in adipose tissue resulting in elevated serum estradiol, may contribute to inhibition of androgen biosynthesis via central feedback mechanism involving the hypothalamus -pituitary gonadal axis [43]. It is well recognized that adipocytes secrete a host of adipokines that regulate a variety of metabolic processes in endocrine, paracrine and autocrine fashion. Thus, adipocytokines, secreted by visceral fat modulate the hypothalamo-pituitary-testicular axis and inhibit T production [44]. Modulation of GnRH secretion by Kisspeptins, produced by adipose tissue causes significant lowering circulating levels of T [45]. Clear and illustrative clinical examples for a role of T in the onset of MetS are demonstrated in patients with PCa undergoing ADT or in men with T2DM. This treatment is accompanied with decreased total lean body mass, increased total fat mass, and, particularly in the longer-term, the development of the MetS. More importantly, acute ADT reduces insulin sensitivity and strongly impairs glycemic control of men with T2DM [46, 47]. Thus, a vicious circle ensues in which MetS suppresses T biosynthesis and conversely, reduced T concentrations predispose and contribute to the onset of development of MetS [32] and in turn obesity.

## OBESITY, LOW TESTOSTERONE AND INFLAMMATION

Visceral obesity is associated with insulin resistance (IR), hyperglycemia, atherogenic dyslipidemia, hypertension as well as pro-thrombotic and pro-inflammatory states. Adipose tissue is presently considered as an endocrine organ secreting various biochemical factors known as adipokines. The adipokines act *via* paracrine, autocrine and endocrine fashion influencing the metabolism of lipid, glucose homeostasis, and may influence cardiovascular risk factors, such as hypertension, as well as thrombotic and inflammatory processes. Tumor necrosis factor-alpha (TNF-α) and interleukin (IL)-6 are inflammatory markers secreted by adipocytes and contribute to the development of IR [48]. Reduced sex hormone levels are associated with increased inflammatory markers [49-51]. In elderly men, an inverse relationship between plasma T and inflammatory markers was observed [49]. Acute withdrawal of T in young, otherwise healthy hypogonadal patients, caused significant increases in IL-6 and TNF-α two weeks after cessation of T treatment. T treatment in men with lower-than-normal serum T produces reduction in adipokines [50], though these are not observed in a short (three-month) study [51]. Whether the effect of T is via inhibition of differentiation of pluripotent cells into adipocytes or proliferation of pre-adipocytes, thus favoring myogenic differentiation [8] remains to be established. In short term studies, (< 3 months) T administration suppressed adiponectin levels [52] with a potentially adverse effect on CVD risk. In patients with newly diagnosed T2DM, T treatment for one year was associated with an improvement of body composition and increased adiponectin levels [12] (Fig. **2**).

## REDUCTION OF AGE-RELATED DECLINE IN TESTOSTERONE

The age-related changes in neuroendocrine function diminish the efficacy of luteinizing hormone in stimulating Leydig cells [40]. An increasing body of evidence suggests that disease significantly contributes to the age-related decline of T [44, 53]. As shown in Fig. (**2**), changes in lifestyle (diet / exercise), leading to weight loss, might partially prevent or redress decline of androgen levels with aging [12]. Also Roux-en-Y gastric bypass surgery reduced body mass index and increased total and free T levels [54]. Apparently, body fat mass possesses the potential to lower serum T, reversible upon weight loss.

## TESTOSTERONE, ADIPOSE TISSUE AND LIPID METABOLISM 

Sex steroid hormones play an integral role in regulating cellular metabolism, accumulation and distribution of adipose tissues. Estrogens, progesterone and androgen receptors are expressed in adipose tissues. Sex steroid hormones regulate the function of adipose tissues by genomic and non-genomic signaling mechanisms. Activation of the cAMP cascade by sex steroid hormones would activate hormone-sensitive lipase leading to lipolysis in adipose tissues. Their activation appears to be involved in the control of pre-adipocyte proliferation and differentiation. T regulates lineage determination in mesenchymal pluripotent cells by promoting the myogenic lineage and inhibiting the adipogenic lineage [8]. T inhibits triglyceride uptake and lipoprotein lipase activity and causes a more rapid turnover of triglycerides in subcutaneous abdominal adipose tissue and less in femoral fat and, maybe, mobilizes lipids from the visceral fat depot [55].

## TREATMENT OF OBESE MEN WITH TESTOSTERONE: EFFECTS ON BODY COMPOSITION

T is an important factor in the etiology of obesity, MetS and its associated diseases T2DM and atherosclerotic disease. The question arises whether T treatment in obesity and MetS and its sequels, such as T2DM and CVD is beneficial. There is increasing body of evidence indicating a beneficial effect of T treatment on visceral fat and other components of the MetS [56]. As shown in Tables **1** and **2**, T administration improves many parameters associated with obesity and effects on lean body mass.

**Fig. (1):**
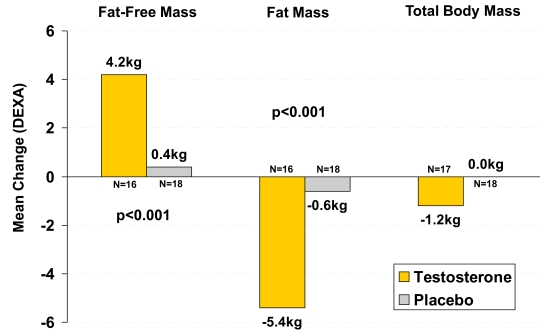
1 year testosterone therapy with parenteral testosterone undecanoate improves body composition in elderly men. Adapted from reference [71].

**Fig. (2):**
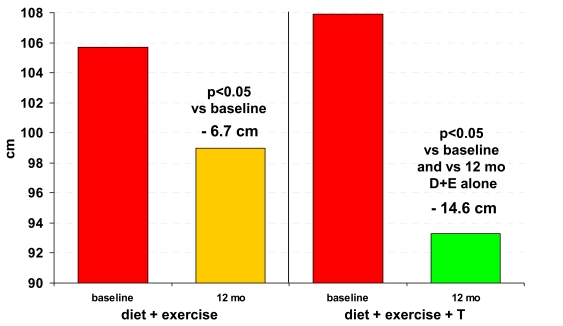
Waist circumference in 32 men with newly diagnosed type 2 diabetes in the DIMALITE (Diabetes Management by Lifestyle and Testosterone) study. Adapted from reference [12].

Changes in visceral fat appear to be related to changes in serum total T [57]. A number of randomized controlled trials have confirmed the beneficial effects of T on body composition and variables of the MetS. In a study of weekly administration of T enanthate 100 mg i.m., there was a significant increase in lean body mass, with a mean change in fat mass of 0.2 kg (n.s), and a decline of serum cholesterol. In an eight months study of 23 middle aged abdominally obese men, a decrease of visceral fat mass (without a change in body mass, subcutaneous fat mass or lean body mass) was observed. The net change of fat mass was -1.8 kg (-6.3%) (n.s.). IR improved and blood glucose, diastolic blood pressure and serum cholesterol decreased with T treatment [58]. In a study of 108 men over 65 years of age fat mass decreased (-2.9 kg, -11.9%; (p<0.001)) and lean mass increased subsequent to T treatment [59]. Similar results were obtained in other studies [60-68]. In a study of 70 men over 36 months, T treatment increased lean body mass, decreased fat mass (-4.5 kg; -16%), decreased total cholesterol, low-density lipoprotein, and leptin [69]. A reduction in leptin and also of adiponectin was found upon T administration to men with TD with or without T2DM [51]. T therapy reduced waist circumference (-1.63 ± 0.71 cm, p = 0.03) and waist/hip ratio (-0.03 ± 0.01, p = 0.01), improved IR and glycemic control in men with TD and T2DM [51, 70]. T treatment reduced fat mass (-5.4±2.4 kg) and abdominal adipose tissue and increased fat-free mass in a one-year study [71] (Fig. **1**). T treatment selectively lessened visceral fat accumulation without change in total body fat mass and increased total body fat free mass and total body and thigh skeletal muscle mass in a study of men over 55 years over 52 weeks [57]. Interestingly, a monthly cycled T regimen using half the T dose compared to the standard care of continuous therapy, improved body composition and increased muscle strength similarly to continuous T [72].

A study of T administration in 207 men over 6 months reported an increase in lean body mass and a decrease in fat mass (-1.3 kg; range -1.8 to 0.8) and a decrease in fat mass percentage (-1.7%, range -2.1—1.1) [10]. The above studies have been conducted in men with low serum T but there are two studies demonstrating a beneficial effect of T administration in men whose serum T is not subnormal but in the low range of normal [57, 58]. In a double blind, placebo-controlled study over a 6-month period, transdermal T treatment was associated with beneficial effects on insulin resistance, total and LDL-cholesterol, lipoprotein(a), and sexual health in hypogonadal men with type 2 diabetes and/or MetS [73].

**Fig. (3):**
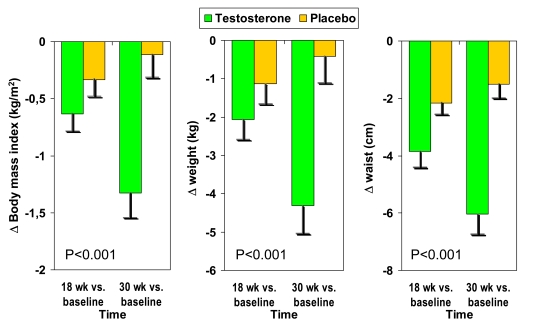
Absolute changes in anthropometric parameters in the double-blind, placebo-controlled Moscow study in 184 men with metabolic syndrome. Adapted from reference [74].

Characteristics of the obese person co-determine the effects of T administration. BMI was significantly associated with the benefit of T treatment on all variables measured except on C-reactive protein [74, 75]. Waist circumference also showed these associations, but BMI appeared to be a slightly stronger predictor than baseline waist circumference (Fig. **3**), it is of utmost interest to indicate that in all three anthropometric parameters there is a progressive improvement in the group of men treated with T. In contrast, the placebo group shows initial improvement after 18 weeks which returns toward baseline levels only 12 weeks later, indicating the transient effect of the physician’s advice to improve life style [74].

## TESTOSTERONE, DIET AND EXERCISE AND WEIGHT LOSS 

The successes of interventions to achieve long-term weight loss in obese men are conflicting and most often disappointing. In a systematic review and meta-analysis of subjects of 60 years or above a modest but significant weight loss of 3.0 kg [95% confidence interval (CI) 5.1-0.9] at 1 year was noted [76]. Another very thorough review reported weight loss in various populations of between 0.04 and 4.9 kg [77]. Total cholesterol did not show a significant change: -0.36 mmol/l (95% CI -0.75 to 0.04). There was no significant change in high-density lipoprotein, low-density lipoprotein or triglycerides. In one study, recurrence of hypertension or cardiovascular events was significantly reduced (hazard ratio 0.65, 95% CI 0.50-0.85). Six-minute walk test did not significantly change in one study. Health-related quality of life significantly improved in one study [70] but did not improve in a second study [73]. The expectations for weight loss in overweight patients are several hundred percent higher than achieved in real life. All these studies did not include baseline T levels as an inclusion/exclusion criterium neither variation from baseline as an endpoint.

Weight loss interventions without changes in aerobic capacity do not appear to enhance muscle oxidative capacity in obesity. There is an increased capacity of intracellular fatty acid transport in skeletal muscle cells in the physiological adaptations of fat metabolism to energy restriction in obesity. In obesity-related IR, the metabolic capacity of skeletal muscle appears to be organized towards fat esterification rather than oxidation and dietary-induced weight loss does not correct this disposition [78]. Mitochondrial electron transport chain (ETC) activity in skeletal muscle in previously sedentary obese men and women was substantially improved by an intervention of moderate weight loss and moderate intensity of physical activity. These biochemical changes in skeletal muscle mitochondria occurred in conjunction with improvement in substrate metabolism and insulin sensitivity in muscle. Interestingly, the increase in ETC activity in skeletal muscle in these previously sedentary obese men and women was greater than that of mitochondrial DNA content, signifying a more pronounced effect on functional capacity of mitochondria than on proliferation [78, 79]. Diet-induced weight loss significantly decreases muscle mass in older adults. However, the addition of moderate aerobic exercise to intentional weight loss attenuates the loss of muscle mass [78]. In a systematic review assessing the effects of energy restriction and exercise on fat-free mass in overweight and obese middle-aged and older adults, it was concluded that exercise is an effective tool in men and postmenopausal women aged ≥ 50 years, with a BMI greater than 25 kg/m^2^ to preserve fat free mass and to combat sarcopenic obesity after moderate weight loss induced by energy restriction [80]. A low-glycemic diet with exercise may provide an alternative and more effective treatment for IR in older obese adults [81, 82]. Exercise training improves resting substrate oxidation and creates a metabolic milieu that appears to promote lipid utilization in skeletal muscle, thus facilitating a reversal of IR [82, 83]. Restoring T to physiological values in men with TD results in loss of fat mass and increase in lean body mass. As a result, the loss of body weight may be quantitatively less impressive. A meta-analysis of randomized controlled trials evaluating the effects of T administration to middle-aged and ageing men on body composition showed a reduction of 1.6 kg (CI: 2.5--0.6) of total body fat corresponding to -6.2% (CI: 9.2--3.3) variation of initial body fat, an increase in fat free mass of 1.6 kg (CI: 0.6--2.6), corresponding to +2.7% (CI: 1.1--4.4) increase over baseline and no change in body weight [83]. In studies with a similar design comparable results were reported with regard to total body fat [64-67, 70, 84]. In a placebo-controlled study using long-acting T undecanoate injections, the reduction of fat mass was 5.4 kg with an increase of lean mass of 4.2 kg [71]. T also reduced total cholesterol by 0.23 mmol/l (CI: -0.37 to -0.10), especially in men with lower baseline T concentrations, with no change in low-density lipoprotein (LDL)-cholesterol. Low serum T levels are associated with an adverse metabolic profile including IR, and there is evidence for a unifying mechanism that low T levels and impaired mitochondrial function promote IR in men [41]. Indeed, administration of T to men with TD improves insulin sensitivity. A study in men with TD and T2DM showed that T replacement also improves glycemic control although this study was non-blinded. In another recent study of T administration in men with MetS and lower-than-normal T levels a reduction in waist circumference, visceral fat mass, and improvement in Homeostasis Model of Assessment - Insulin Resistance (*HOMA*-*IR*) without changes in body mass index was observed [70, 85].

T may exert a dual effect on metabolism with an acute improvement in insulin sensitivity that occurs rapidly within a few days to few weeks of treatment, and before loss of fat mass becomes evident; and a prolonged effect achieved when a significant reduction of total and visceral body fat occurs [86, 87]. Given the fact that T replacement will promote a more active life-style, the rapid effects are needed to prepare the body to greater energy expenditure. In fact, physicians treating obesity should be aware of the risks of recommending physical exercise or intensive training to obese men with TD. In view of the importance of T in bone and muscles homeostasis, it is evident that obese men should receive androgen replacement prior to commencing physical training [88].

## SAFETY OF TESTOSTERONE THERAPY 

Studies to prove long-term safety of T have not been performed and will not be performed shortly because of logistics and budgetary problems. Physicians hesitate to prescribe T to men over the age of 50 years fearing it will induce PCa. Recent findings have placed such fear in a more rational perspective. T treatment in men [89] did not exacerbate voiding symptoms due to benign prostatic hyperplasia. The occurrence of PCa after T administration in (elderly) men has been reported, mostly in case reports [90]. By contrast, a host of studies, using various designs and T formulations, over periods ranging from several months to 15 years, in men with a wide range of ages, have not revealed an increased risk of PCa [91]. A meta-analysis found that T treatment in older men compared to placebo was not associated with a significantly higher risk of detection of PCa [89], although the frequency of prostate biopsies was much higher in the T-treated group than in the placebo group [89]. There is no convincing evidence that T is the main factor in the development or progression of PCa in men [92, 93]. But as indicated above, the definitive studies to define the safety of T have not been carried out. Guidelines for monitoring have been developed which, if rigorously applied, render T treatment to be a safe therapy in men without (a prior history of) PCa. 

T produces a dose dependent effect on hemoglobin and hematocrit values [94], apparent in a number of studies , and remarkably, this effect is more pronounced in older men [95-97]. Though an elevated hematocrit is associated with stroke, and coronary heart disease, a relation between increased hematocrit as a result of androgen supplementation as such and an increased risk for stroke or any cardiovascular event in general has not been demonstrated by large meta-analyses of placebo-controlled trials of T administration to (elderly) men [14, 89]. Polycythemia is a manageable risk of androgen administration when hemoglobin levels and hematocrit are monitored and the dose of T is adjusted.

Until a decade ago, it was a widely held belief that androgens have an atherogenic effect and thus led to CVD, and androgen therapy was regarded a risk of developing CVD. Over the last decade several studies have examined the relationship of androgens with CVD and concluded that it is no longer tenable to regard T as a culprit in the etiology of CVD [98, 99]. The study of Basaria *et al. *[100] has drawn new attention to the potential cardiovascular risks of T administration to elderly frail men, but the study has also been critically examined for a number of potential biases, addressed in a recent review [101]. 

T therapy in elderly men may be viewed as a responsible practice provided certain guidelines produced by professional scientific and clinical organizations are followed with regard to T therapy in elderly men [16]. This consensus is based on expert opinion and, therefore, the need for longer-term safety studies remains urgent. 

## FUTURE PERSPECTIVE

**Fig. (4):**
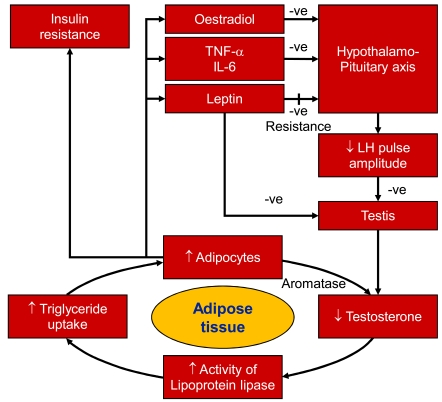
The vicious cycle of testosterone deficiency and obesity. Adapted from Jones TH. Pract Diab Int 2007; 24(5): 269-277.

Obesity is a growing health problem worldwide. It impairs health and thus quality of life. Treatment is far from simple. Obesity produces a host of metabolic changes at the molecular and cellular levels in a host of tissues, including the skeletal muscle. These metabolic changes bring about programmed cell death and increased inflammatory markers and produce a vicious cycle that contributes to TD and obesity (Fig. **4**). The link between IR, obesity and MetS and the onset of T2DM is well established, however the exact cellular mechanisms remain under extensive investigations.

Diet treatment is only marginally successful and leads to loss of muscle mass, which may be prevented by combining diet with exercise. Although treatment of obesity by lifestyle interventions in high risk patients, especially those at high risk for T2DM is highly recommended, it is usually little successful and of limited clinical benefit. Pharmacological treatment is only modestly successful and not without cardiovascular side effects [102]. A substantial number of obese men with T2DM exhibit reduced androgen levels. In these subjects, T treatment reduces fat mass and increases lean body mass and very likely also improves insulin sensitivity. T treatment improves muscle mass, reduces fat mass and muscle strength and physical performance. In addition, T increases motivation and decreases fatigue [103] and may therefore enable men to better adhere to an exercise programme. Thus, T may represent a useful tool in managing obesity in hypogonadal men when used together with diet and exercise. 

There is as yet a lack of definition of a level of testosterone for initiating safe supplementation therapy in patients who are obese but do not suffer from testosterone deficiency symptoms. It is of note that in aging men, psychosomatic complaints and metabolic risk relate to levels of testosterone in a symptom-specific manner; some symptoms appear above the lower cut-off levels for the reference range of T [104]. But, until more specific information is available, T treatment should be limited to men with established biochemical criteria of TD [15, 16]. A recent study provided quantitative information. The mean (SD), median (quartile), and 2.5th percentile values were 723.8 (221.1), 698.7 (296.5), and 348.3 ng/dl for total T and 141. 8 (45.0), 134.0 (60.0), and 70.0 pg/ml for FT, respectively, clinically validated by the fact that in all three samples, men with low TT and FT were more likely to have slow walking speed, difficulty climbing stairs, or frailty and diabetes [15]. But beneficial effects on features of the metabolic syndrome have been encountered in men with serum T levels above the lower limit of reference values [57, 105]. 

T treatment has positive effects on a number of parameters of cardiovascular health, such as serum LDL-cholesterol, blood pressure and heart rate [98]. Physicians managing overweight patients and its sequels (T2DM, CVD) are either unfamiliar or unaware of the physiology and pathophysiology of TD and its impact on body composition, the potential of T treatment in men with MetS and its sequels is not seriously considered as an option. Although comparison studies are lacking, the magnitude of changes obtained with T replacement therapy appear superior (or at least as effective as) most drugs used to reduce excess fat mass. A significant aspect of treating men with TD is the mood-elevating action of T in men with MetS who are often depressed and dysphoric. This aspect may bolster motivation to lose weight by adhering to a regimen of diet and exercise. Finally, recommending exercise to men with TD, for weight reduction, may expose these patients to severe risks [88]. To establish T therapy as a legitimate medical tool in the management of obesity in men with TD, more specific studies will be required by the regulatory agencies. These studies should convincingly demonstrate that abdominal obesity and MetS are ameliorated by normalizing T levels in hypogonadal men. Such studies should be up to the level of at least phase three to assess the efficacy with more narrow confidence intervals and to provide long-term safety data.

## Figures and Tables

**Table 1.** Changes in Parameters of Obesity Upon Testosterone (T) Treatment Versus Placebo (Plc).

Reference/Parameter	Preparation	Treatment Duration	Change T	Change Plc	Net Change
***Total fat mass (kg)***					
Marin 1993 [58]	Gel	9 mo	-1.8	0.6	-2.4
Snyder 1999 [59]	Patch	36 mo	-3.3	-1.3	-2.0
Kenny 2001 [62]	Patch	12 mo	-1.7	0.3	-1.4
Ferrando 2002 [63]	TE	6 mo	-3.6	0.3	-3.9
Boyanov 2003 [64]	Oral TU	3 mo	-1.65	-0.25	-1.4
Crawford 2003 [65]	Mixed esters	12 mo	-2.3	0.7	-3.0
Steidle 2003 [66]	Gel	3 mo	-0.8	-0.1	-0.7
Steidle 2003 [66]	Patch	3 mo	-0.4	-0.1	-0.3
Wittert 2003 [61]	Oral TU	12 mo	-0.2	0.85	-1.05
Casaburi 2004 [67] (no tr.)	TE	10 wk	-1.01	-0.08	-0.93
Casaburi 2004 [67] (training)	TE	10 wk	-1.41	-0.13	-1.28
Svartberg 2008 [71]	Inj TU	12 mo	-5.4	-0.6	-4.8
Allan 2008 [57]	Patch	12 mo	-0.5	0.1	-0.6
Emmelot-Vonk 2008 [10]	Oral TU	6 mo	-1.0	-0.1	-0.9
Srinivas-Shankar 2010 [68]	Gel	6 mo	-0.8	-0.3	-0.5
***Visceral adipose tissue (kg)***								
Marin 1993 [58]	Gel	9 mo	-0.6	0.2	-0.8
Allan 2008 [57]	Patch	12 mo	-0.2	0.5	-0.7
***Trunk fat (kg)***					
Casaburi 2004 [67] (no tr.)	TE	10 wk	-0.55	0.34	-0.89
Casaburi 2004 [67] (training)	TE	10 wk	-0.67	0.11	-0.78
Page 2005 [69]	TE	36 mo	-1.9	-0.4	-1.5
Allan 2008 [57]	Patch	12 mo	0.1	0.0	0.1
***Visceral adipose tissue (cm^3^)***					
Svartberg 2008 [71]	Inj TU	12 mo	-38	-11	-27
***Subcutaneous adipose tissue (kg)***					
Marin 1993 [58]	Gel	9 mo	-1.2	0.5	-1.7
Allan 2008 [57]	Patch	12 mo	-0.1	0.0	-0.1
***Subcutaneous adipose tissue (cm^3^)***					
Svartberg 2008 [71]	Inj TU	12 mo	-49	-10	-39
***Total adipose tissue (cm^3^)***					
Svartberg 2008 [71]	Inj TU	12 mo	-86	-27	-59
***Right leg fat (kg)***					
Page 2005 [69]	TE	36 mo	-0.9	0.1	-1.0
***Percentage total body fat (%)***					
Sih 1997 [84]	TC	12 mo	-1.9	19.3	-21.2
Boyanov 2003 [64]	Oral TU	3 mo	-3	-0.1	-2.9
Crawford 2003 [65]	Mixed esters	12 mo	-10.9	3.4	-14.3
Steidle 2003 [66]	Gel	3 mo	-1.2	-0.2	-1.0
Steidle 2003 [66]	Patch	3 mo	-0.5	-0.2	-0.3
Casaburi 2004 [67] (no tr.)	TE	10 wk	-6	-0.1	-5.9
Casaburi 2004 [67] (training)	TE	10 wk	-9.4	-2.2	-7.2
Page 2005 [69]	TE	36 mo	-17.0	1.0	-18.0
Kapoor 2007 [51]	Mixed esters	3 mo	-3.7	-1.5	-2.2
Kapoor 2006 [70]	Mixed esters	3 mo	-3.0	-1.8	-1.2
Svartberg 2008 [71]	Inj TU	12 mo	-18.9	-1.9	-17.0
Allan 2008 [57]	Patch	12 mo	-2.9	0.4	-3.3
Emmelot-Vonk 2008 [10]	Oral TU	6 mo	-4.7	0.0	-4.7
Aversa 2010 [85]	Inj TU	24 mo	-18.5	0.5	-19
Aversa 2010 [87]	Inj TU	12 mo	-18.4	0.6	-19.0
***Waist circumference (cm)***					
Marin 1993 [58]	Gel	9 mo	-2.5	-0.6	-1.9
Kapoor 2007 [51]	Mixed esters	3 mo	-2.0	0.1	-2.1
Kapoor 2006 [70]	Mixed esters	3 mo	-1.6	N/A	N/A
Svartberg 2008 [71]	Inj TU	12 mo	-3.0	-1.0	-2.0
Heufelder 2009 [12]	Gel	12 mo	-14.6	-6.7	-7.9
Aversa 2010 [85]	Inj TU	24 mo	-8.5	-0.5	-8.0
Kalinchenko 2010 [74]	Inj TU	30 wk	-5.8	-1.5	-4.3
Aversa 2010 [87]	Inj TU	12 mo	-8.7	1.1	-9.7

Gel: testosterone gel Patch: testosterone patch TE: parenteral testosterone enanthate Oral TU: oral testosterone undecanoate Mixed esters: mixed parenteral testosterone esters Inj TU: parenteral testosterone undecanoate TC: parenteral testosterone cypionate

**Table 2.** Changes in Lean Mass Upon Testosterone (T) Treatment Versus Placebo (Plc).

Reference / Parameter	Preparation	Treatment Duration	Change T	Change Plc	Net Change
***Total lean mass (kg)***								
Marin 1993 [58]	Gel	9 mo	1.5	0.2	1.3
Snyder 1999 [59]	Patch	36 mo	2.1	-0.3	2.4
Kenny 2001 [62]	Patch	12 mo	1.0	0.2	0.8
Crawford 2003 [65]	Mixed esters	12 mo	1.8	-0.5	2.3
Ferrando 2003 [63]	TE	6 mo	4.2	-2	6.2
Steidle 2003 [66]	Gel	3 mo	1.7	0.6	1.1
Steidle 2003 [66]	Patch	3 mo	0.9	0.6	0.3
Wittert 2003 [61]	Oral TU	12 mo	1.0	-1.0	2.0
Casaburi 2004 [67] (no training)	TE	10 wk	2.3	-0.21	2.51
Casaburi 2004 [67] (training)	TE	10 wk	3.29	0.2	3.09
Page 2005 [69]	TE	36 mo	3.7	0.0	3.7
Kapoor 2006 [70]	Mixed esters	3 mo	1.23	0.67	0.56
Svartberg 2008 [71]	Inj TU	12 mo	4.2	0.4	3.8
Allan 2008 [57]	Patch	12 mo	0.8	-0.3	1.1
Emmelot-Vonk 2008 [10]	Oral TU	6 mo	1.1	-0.3	1.4
Srinivas-Shankar 2010 [68]	Gel	6 mo	1.1	-0.2	1.3
Aversa 2010 [85]	Inj TU	24 mo	4.8	-0.1	4.9
Aversa 2010 [87]	Inj TU	12 mo	4.2	-0.6	4.8

Gel: testosterone gel Patch : testosterone patch TE: parenteral testoterone enanthate Oral TU: oral testoterone undecanoate Mixed esters: mixed parenteral testoterone esters Inj TU: parenteral testoterone undecanoate

## LIST OF ABBREVIATIONS

ADT: = Androgen Deprivation Treatment
BMI: = Body mass Index
CI: = Confidence Interval
CVD: = Cardiovascular Disease
ED: = Endothelial Dysfunction
ETC: = Electron Transport Chain
FA: = Fatty Acids
HbA_1c_: = Glycated Hemoglobin
IL-6: = Interleukin 6
IR: = Insulin Resistance
LH: = Luteinizing Hormone
MetS: = Metabolic Syndrome
MMAS: = Massachusetts Male Aging Study
MRFIT: = Multiple Risk Factor Intervention Trial
NHANES III: = Third National Health and Nutrition Survey
PCa: = Prostate Cancer
SHBG: = Sex Hormone Binding Globulin
T: = Testosterone
TD: = Testosterone Deficiency
TNF-α: = Tumor Necrosis Factor-alpha
T2DM: = Diabetes Mellitus Type 2
WC: = Waist Circumference
WHR: = Waist/Hip Ratio
